# Single-Sensor Vibration-Scanning Method for Assessing the Mechanical Properties of 3D Printed Elements

**DOI:** 10.3390/ma14051072

**Published:** 2021-02-25

**Authors:** Ryszard Buchalik, Grzegorz Nowak

**Affiliations:** Department of Power Engineering and Turbomachinery, Silesian University of Technology, Konarskiego 18, 44-100 Gliwice, Poland

**Keywords:** 3D printing, additive manufacturing, material properties, phase shift measurement, vibration scanning, resonance, vibration shapes

## Abstract

This paper considers issues related to the assessment of the mechanical properties of elements made with 3D printing technology. To enable experimental testing, an automated test stand was built to perform amplitude and phase angle measurements of any point of the specimen. A contactless, optical measurement method was selected, as it is especially adequate when it comes to elements with small dimensions and masses. One innovative element of the test stand is the original method of phase angle measurement using a single vibration sensor fitted with a system forcing and ensuring full measurement synchronization and dynamic state repeatability. Additionally, numerical models of tested objects were produced and simulations of their oscillations were performed. Based on that, the properties of the tested material (PLA) were considered, with a special focus on the density, elastic modulus, and damping. The analyses were conducted for a few elements with different dimensions at different vibration frequencies.

## 1. Introduction

In recent years, there has been a huge increase in interest in Additive Manufacturing (AM) as a modern technology for the production of a wide range of elements. The method’s advantages are the relatively fast production, the possibility of creating complex shapes and joining different materials, and the ease of obtaining the desired color or finish [1]. The use of varied internal structures in this technology makes it possible to shape the properties of produced elements, which is extensively presented in [2]. Gibson et al. [3] describe the application of bulk metallic glasses in AM, which behave like thermoplastics. Considering the recent development of 3D printing technology and the increasing potential of the technology’s application in many different fields, it is important to establish the mechanical properties of elements made in this way [4,5]. There are works describing certain features and mechanical (material) properties of such materials [6,7,8,9,10,11,12], including product documentation of the raw material supplied by the manufacturer. However, most of them primarily concentrate on testing the material in steady states and on slow changes in essential parameters, such as the load. They offer very scarce information on the material behavior under fast-changing loads (a few dozen Hz and more), i.e., vibration. In this area, work focuses on the impact of geometric discontinuities (e.g., cracks) on the dynamic state of the element [13,14]. The technical data supplied by the manufacturers of devices, on the one hand, and filament producers, on the other hand, are not always confirmed by experimental testing results. Selected examples from the literature focus on the impact of the infill and the printing direction [15,16], taking account of fatigue [17] and creep [18,19] processes, the printing system vibrations [20] and damping [21]. Testing materials made with Additive Manufacturing technology is onerous due to the substantial impact of a great number of factors. The finished product parameters vary, depending on a number of parameters of the production process. The produced object’s mechanical parameters depend, in a complex way, on the temperature of the nozzle depositing the material, the method of the material feeding, the shape and diameter of the nozzle and the nozzle thermal conductivity, the ambient temperature, the cooling method and intensity, the layer thickness, the printing speed, the material storage conditions (the history of temperature, time, and humidity), the printer accuracy, and the whole printed element cooling rate. Another key component of the 3D printing system that has an essential impact on the final product parameters is the program creating the extruder movement trajectory [22]. In the case of fused filament fabrication technology, an enormous number of combinations of printing parameters are used on the shell and in the infill. The parameters of the first layer (in contact with the base platform) also differ from the other layers. An element made with this technology is usually characterized by strongly anisotropic properties, which is mainly due to the existence of layers and contact between them [15,16]. An essential part of the production process is to determine the spatial orientation according to which the object will be made. Material cooling also generates internal stresses and may result in bending of the finished product [23]. 

The behavior of a mechanical element when exposed to vibrations and the possibility of resonant frequency excitation within it can lead to serious problems in the design and operation of machines [24]. There are studies based on the vibration analysis of elements made with 3D printing technology [25,26,27,28,29,30,31]. Some of them focus on determining damping inside the material if oscillating motion occurs and make use of comparative analysis. An exhaustive theoretical description of the phenomenon is presented in [26]. Gietl et al. [21] presented the way in which the impact of the medium (air resistance) is eliminated from measurements using a fiber-optic sensor. Efforts aiming to establish material parameters seem especially important when it comes to design and prediction of the occurrence of potentially harmful resonances of elements made with 3D technology. They will make it possible to simulate this type of system at the prototype-making stage and avoid many operating problems, e.g., excessive wear, noise, fatigue-related damage, etc. It is worth mentioning that very technologically advanced elements, even including turbine blades, can also be successfully produced with AM technology [32]. Due to the progress in the field of searching for materials with high vibration-isolating capabilities [33], it is also important to search for new methods for determining the material’s elasticity and ability to dissipate the energy (damping) of its vibrations (damping), characterized by simplicity and the possibility of application in various conditions. The description of material parameters is additionally complicated in the case of structures reinforced with fibers made of different materials and embedded within the base material [34]. Damping or elasticity calculations can be very complex for specimens composed of many different material layers characterized by different properties [35] and different joints or infill [36]. The destruction of material due to vibration shapes, among other factors, can be taken into account [37].

In connection with the above-described aspects of AM technology and its expected development, vibration testing methods should be widely used. Typically, vibration testing involves the use of a sensor rigidly and directly mounted on the tested object. The only more widespread type of non-contact vibration sensor is the inductive sensor; however, its use is practically only possible in the case of materials showing magnetic properties, with a significantly different electrical and magnetic permeability from a vacuum. The only rational choice in the case of AM materials with a relatively low density that are light, small, and non-magnetic seems to be the optical measurement method. Due to the significant technological progress and reduction of the costs of photosensitive and laser elements, it seems reasonable to develop optical methods for vibration measurement. 

There are several basic methods of optical vibration measurement, which allow various physical quantities to be measured. The first may be displacement-triangulation sensors in many arrangements [38,39] or an analysis of the image of the object on which the appropriate pattern is displayed at defined angles [40,41,42]. Due to the differential-integral dependence, the mechanical quantities can be mutually converted. However, it should be remembered that the numerical accuracy of the result changes, depending on the frequency of vibrations. 

The aim of this paper is to consider issues related to the mechanical properties of objects made with 3D printing technology. An analysis of selected mechanical parameters is presented based on dynamic testing and the use of resonance frequencies. Experiments were performed to this end using a novel method of vibration measurement on a purpose-made measuring stand. The analysis covers the vibration amplitude and the phase shift by means of an unconventional method using only one displacement measuring sensor. Experimental data collected in this way can be coupled with a sophisticated numerical method [43] to determine the material properties and their uncertainty. The method proposed below can be applied to specimens made from different kinds of materials, even those which are naturally non-isotropic and non-uniform [44]. 

## 2. Materials and Methods

A purpose-designed test stand was constructed to perform measurements of dynamic states of specimens made with 3D printing technology.

### 2.1. Test Stand

**Figure 1:**
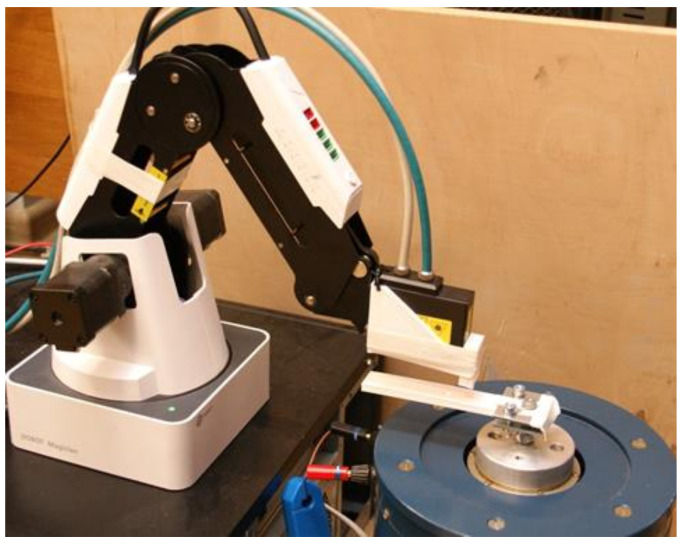
Test stand general view.

**Figure 2:**
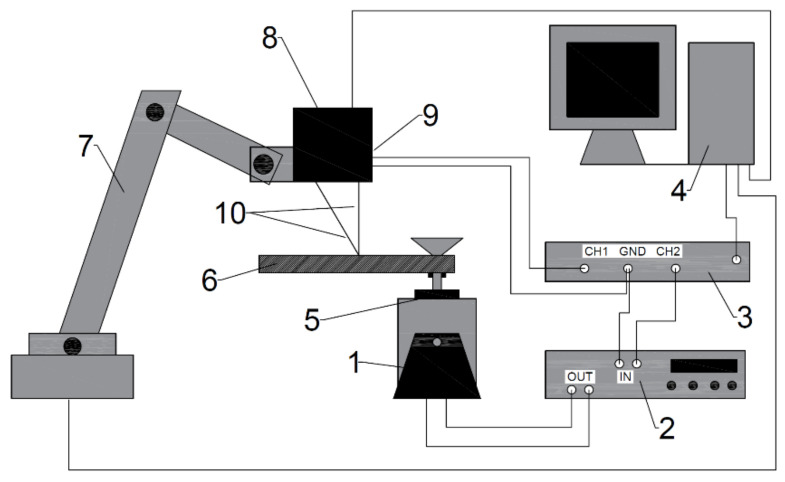
Diagram of the test stand: 1—vibration exciter; 2—amplifier; 3—digital-to-analog converter; 4—computer; 5—mounting; 6—specimen under investigation; 7—robotic arm; 8—optical sensor (distance measurement); 9—synchronizing signal connection; and 10—laser beam.

The test stand (Figure 1 and Figure 2) is composed of a vibration exciter and a measuring system; the whole stand is computer-controlled. The measuring system’s fundamental element is a triangulation displacement sensor. Its operating principle is the emission of a laser beam illuminating the investigated point of the specimen; the beam is then subjected to diffusive reflection. The reflected signal returns to the sensor at an angle, depending on the reflection point distance from the sensor, which is recorded by a light-sensitive matrix (one-dimensional image sensor) placed in the instrument and fitted with an appropriate optical system (lenses). The optical sensor returns a digital string of data representing the signal sampled with the frequency of 48 kHz and resolution reaching a few micrometers in the operating range of 10 mm. The test stand also includes a robotic arm to which the above-mentioned sensor is fixed. The arm enables the full control of motion in three spatial directions.

The analysed specimen is fixed to the electromagnetic core of the vibration exciter powered by an analogue amplifier. The signal is generated using the first channel of the computer sound card. The second channel is used to generate a rectangular waveform (with the frequency of 48 kHz), which is the synchronizing signal for the optical sensor. In this case, each ascending slope triggers a recording of the observed point distance from the sensor. The entire system is operated, programmed, and controlled in the LabView^®^ environment, including the readout and processing of measurement quantities (LabView^®^ Sound and Vibration Toolkit). After the measurement points and the range of tested frequencies are determined, the measurement is fully automatic. The stand’s basic operating mode is vibration scanning of the specimen. This process requires, firstly, an array containing tested frequencies (usually, a range of values with monotonic, arithmetic, or geometric progression is determined) and, secondly, the coordinates defining all points of measurement (packets of three numbers—x,y,z). The number of individual measurements is the product of the number of geometrical locations and the number of scanned frequencies. Such a single measurement consists of placing the sensor in a set location and starting to send a signal with a set frequency to the input of the electromagnetic exciter amplifier, which is thus set in motion. At the same time, the exciter core acts as the tested specimen support. The motion of the support is thus the base excitation with an amplitude and frequency set by the user. After the exciter is activated, it is necessary to bring the oscillating system to a quasi-steady state, which, as demonstrated by the tests, takes a few seconds, depending on the vibration frequency. At this stage, the synchronizing signal is flat—no data are collected. Then, the next stage of collecting data from the optical displacement sensor begins, at which point the synchronizing signal starts to oscillate at 48 kHz. The data collection time should be long enough to capture many full oscillation periods (being the effect of the input function frequency), including potential occurrence of acoustic beats. An appropriately long measurement time, i.e., a period producing an adequately large set of measuring data, enables correct data processing and, first of all, makes it possible to improve the accuracy by averaging data for many periods. Based on the testing results, the duration of the data acquisition stage adopted in this study was one second. After the measurements are completed, the signal forcing the exciter motion and the sensor synchronization is turned off.

The number of collected data defining the measurement point distance from the sensor should be equal to the product of the sampling frequency (48 kHz) and the measuring time. The obtained set of measurement results is then processed. First, the number of collected data is checked. It is also checked whether each data value is included within the measuring range and whether there are no transmission errors. In the next step, the data are filtered using a band-pass filter to capture the one and only frequency-dependent component of the currently tested quantity (equal to the input function supplied to the vibration exciter). Fourier analysis is then conducted and the main frequency is determined, together with its amplitude and phase. It is also confirmed whether it is equal to the input function frequency (with a small error margin taking account of the numerical character of the testing and the finite nature of the data set). 

In addition, methods allowing compensation of the elasticity of the specimen holder may be used [45]. The proposed arrangement of the test stand may also be employed for imaging and determining significant characteristics for nonlinear elements [46]. After minor modifications, it can also be applied to investigate complicated models made on a small scale, especially damping in the structure [47].

### 2.2. Phase Angle Determination Procedure

**Figure 3:**
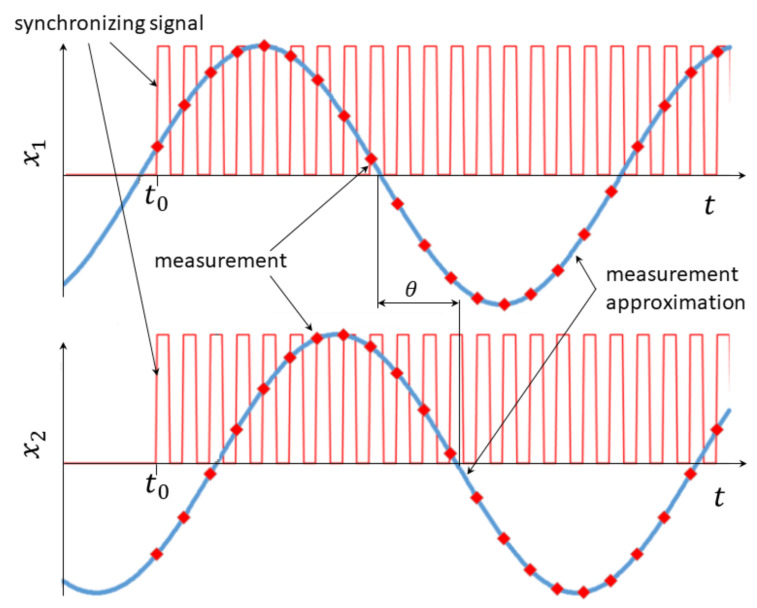
Schematic diagram of the phase shift measurement method.

The most innovative part of the constructed test stand is the possibility of measuring the phase angle on a test rig fitted with a single measuring sensor. The process makes use of the mutual relative repeatability of the input function signal and of the signal synchronizing the measuring sensor (and also of the signal triggering data acquisition) described above in Section 2.1. Data collection always begins precisely at the same instant of the vibration exciter cycle defined by the base excitation waveform, i.e., the measurement always begins at the same moment of the cycle duration ($t_{0}$), at a set value of the input function frequency. Consequently, resultant signals (the time-dependent phase shift curve) collected at two points, or more specifically, their relative phase shift, represent the real phase shift between oscillating motion at these points at a set frequency. The idea of measurement procedure realization is illustrated in Figure 3. The top and bottom waveforms represent the measurement results for two different points on the specimen. The blue line marks the history of the actual time-dependent displacement of the tested point on the specimen. The red line illustrates the synchronizing signal, the beginning of which is synchronized with the input function—it always appears at the same moment of the cycle, and consequently, at the same position of the exciter core, and hence, at the same position of the entire specimen. The red markings denote collected measurement data, based on which, after they are connected, the phase shift θ can be determined. The quantity on the vertical axis in Figure 3 can be expressed as displacement, velocity, or acceleration, which is irrelevant for the phase angle measurement idea. 

The phase angle determination method consists of setting the measuring sensor so that the measuring light beam falls onto the tested element. A two-channel signal is generated next, lasting long enough for the element vibrations to stabilize (usually from a fraction of a second to a few seconds). The signal from one of the channels consists of a sinusoidal waveform starting from zero; the other channel consists of a zero curve (no signal) for a set, precisely measured over time (vibration stabilization). A signal is then generated with a frequency from several to a few dozen kHz, determining the data sampling frequency, i.e., the accuracy of frequency and the phase shift measurement by the measuring sensor. The sampling frequency depends on the maximum frequency of the measuring sensor operation—the higher the sampling frequency, the more accurate the history of oscillations and thus phase angle determination accuracy. The procedure is repeated, varying the measuring sensor location so that the light beam falls onto another point on the tested element.

The measurements result in two one-dimensional tables with numerical values, each of which includes recorded measurements. Plotting their (approximated) history in a chart as a function of time makes it possible to draw a comparison between signals from the two points and determine the phase angle shift between them (θ) Therefore, the essence of the measurement is the use (by controlling the exciter) of fully repeatable base excitation precisely synchronized in time with data acquisition. The key component here is the tested process repeatability—the same history of the input function force is applied to the same system, which results in identical specimen behavior. The measuring sensor synchronizing signal (the moment of the start of the measurement) always appears at the same instant of the period of the exciter vibrations. This makes it possible to subsequently collect data for any number of measuring sensor locations, with a separate measurement being performed for each of these locations. Combining the data collected in this way enables determination of their relative phase shift characteristics. It is assumed that the material properties do not change during the test. 

## 3. Beam Vibration Model

The tests presented in the paper were carried out for a prismatic cantilever beam with a uniformly distributed mass fixed on one end and subjected to dynamic input functions (the Euler–Bernoulli model). Because, during the testing, the oscillating motion measurements are only possible in one (vertical) direction (due to the test bench design) and the beam in question is symmetric, the vibrations may be considered one-dimensional. Only oscillations occurring in this vertical direction are taken into account; torsional forms are ignored, even though they are also present, resulting in slight displacements in the analysed direction. Additionally, oscillations due to deviation from the assumed ideal and symmetrical dimensions of the system are also ignored. Under these assumptions, the analysed specimen reference geometry can be simplified. The analysis only covers the change in vibration values along the specimen length, because a change of the measurement coordinate in other spatial directions should not affect the results—the system can be considered one-dimensional. The oscillating motion direction is perpendicular to the “length” coordinate.

The natural vibration form is determined using a model constructed based on Equation (1). In the determination of the vibration frequency of a prismatic cantilever beam with a constant density along the beam’s entire length, the basic assumption is equilibrium between internal forces (the left side of the equation) and inertia (the right side of the equation) for each element along the beam length (dx):

(1) $$\frac{\partial V_{x}}{\partial x}dx=m\frac{\partial^{2}y}{\partial t^{2}}dx,\;$$

where $V_{x}$ denotes the shearing force; m is the linear mass (mass per length); and x and y are axial and lateral coordinates, respectively.

Considering the relation between the bending moment $M_{x}$ and the shearing force for the prismatic beam,

(2) $$\frac{dM_{x}}{dx}=V_{x}$$

and taking account of the beam deflection equation

(3) $$\frac{M_{x}}{EI}=\frac{\partial^{2}y}{\partial {x^{2}}^{\prime }}\;\;$$

the fourth-order differential equation with respect to time (t) is obtained (4):

(4) $$\;\frac{\partial^{2}y}{\partial x^{2}}+\frac{EI}{m}\frac{\partial^{4}y}{\partial t^{4}}=0,\;\;$$

where E and I are the elastic modulus and the second moment of the area, respectively. In terms of the considerations presented herein, the essential solutions are only those corresponding to standing waves, i.e., cases where natural vibration forms are created. The solution of Equation (4) can therefore be presented as a function of separable variables—a product of two functions where one depends on time and the other only depends on space. 

Consequently, vibration forms can be expressed using relation (5), which is a general solution of differential Equation (4) [48]:

(5) $$X\left(x\right)=C_{1}\sin\left(\alpha x\right)+C_{2}\cos\left(\alpha x\right)+C_{3}\sinh\left(\alpha x\right)+C_{4}\cosh\left(\alpha x\right),\;$$

where X denotes deflection at axial coordinate x, and $\alpha =\sqrt[{4}]{\frac{m\omega^{2}}{EI}\;}$. Constants $C_{n}$ are determined using boundary conditions. Since one end of the beam is fixed (x=0), no motion is possible there (neither translation nor rotation occurs).

**Figure 4:**
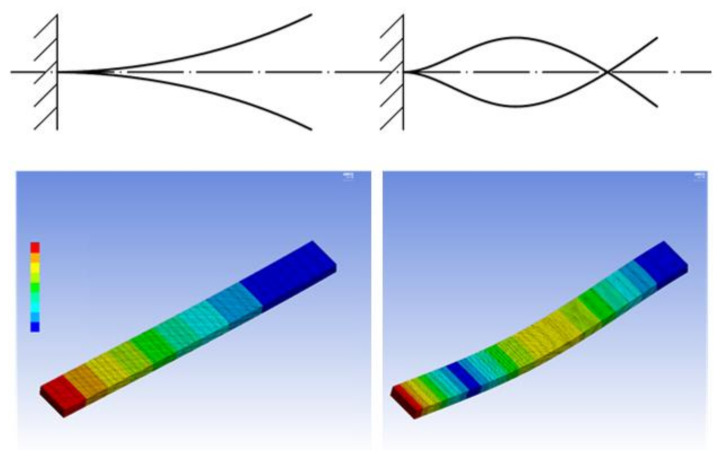
First (**left**) and second (**right**) vibration form in the direction perpendicular to the beam; top: schematic diagram of vibration forms; bottom: FEM results (blue- smallest amplitude, red biggest amplitude).

Moreover, due to free motion of the other end, the forces acting at this point (x=l) are zero, l is the length of the beam. The solution expressing frequencies in this situation is

(6) $$f=\frac{\beta }{l^{2\;}}\sqrt{\frac{EIl}{m}},$$

where β=0.560 and β=3.507 for the first and second bending form under analysis, respectively. The analysed forms of the prismatic cantilever beam natural vibration are presented in Figure 4. 

## 4. Experimental Testing

The tests were performed on specimens in the form of a prismatic cantilever beam with a rectangular cross-section made with Additive Manufacturing technology. The specimens were printed using the Makerbot Replicator 2X device with the following settings: Infill of 100% (printing degree: 45°, moved with every layer by 90°) and two shells. The nozzle temperature was 210 °C, and the printing proceeded horizontally (the shortest side upwards). The Basf Innofil 3D PLA WHITE filament was used. Several specimens with different dimensions in the form of rectangular prisms were made for the purpose of testing. The ones selected for the measurements were as follows: I—197 × 20 × 6 mm; II—197 × 20 × 4 mm; and III—117 × 20 × 4 mm. The outermost 19-mm part of the specimen was slotted into the fixing system made of two flat steel elements. The specimen was clamped in between using screws and the whole specimen was attached to the electromagnetic exciter core. The fixing system (cf. Figure 2) was a compact structure with a much higher stiffness than the tested specimens. The first step was to establish the specimen’s true density using laboratory pan scales. The calculations were performed by adopting the specimen dimensions defined in the geometry source file. The actual measurement of geometry indicated slightly higher values due to the surface texture being an effect of the manufacturing technology (excessive extrusion, material swelling, and excessive looseness). The filament’s technical data sheet [49] lists selected data characterizing its raw state and the printed product material. The density is given as 1.26 g/cm^3^. The measurement results are listed in Table 1. The determined density value resulted from the specimen’s geometrical dimensions and mass. 

The specimen refers to the tested beam part protruding from the fixture. The first to be tested was the 197 × 20 × 6 mm specimen. It is hereinafter referred to as specimen I. To make a rough estimate of its dynamic characteristic, the specimen was modeled using the Ansys Mechanical package. The model only comprises the part protruding from the support, i.e., the tested dimensions are 178 × 20 × 6 mm. The beam was fixed on the 6 × 20 front plane to obtain the cantilever beam model. The established value of density (1.24 g/cm^3^) and the elastic modulus value read from the material specification sheet data (2852 MPa) were applied. Because the geometrical dimensions and mass were measured with the accuracy of 0.1 mm and 0.01 g, respectively, the density determination uncertainty was estimated at the level of 0.03 g/cm^3^. 

### 4.1. Determination of Resonance Frequencies Using the Displacement Amplitude

Following the procedure described in Section 2.1 above, the tested specimens’ displacement amplitude was measured in frequency ranges determined through numerical simulations. The obtained values of frequency (numerical simulation) for subsequent vibration modes are listed in Table 2. The vibration forms for which the motion of every point only occurs in the direction possible to record due to the test bench design (vertical) are marked with an asterisk (*). This direction remains the same, irrespective of changes in the horizontal coordinate normal to the specimen axis. The selected vibration forms therefore represent motion that can be defined using a single coordinate and are a solution of the Euler–Lagrange equation. Mode 2 (Table 2) represents transverse vibration in the second plane (left-right, not analysed); modes 4, 6, and 8 are related to the rotation of cross-sections (specimen torsion); and mode 9 involves simple compression and axial tension of the specimen (longitudinal wave mode). 

In the case of modes used for further analysis (modes 1 and 3 from Table 2), the selection of the Poisson ratio, and consequently, of the shear stiffness (the Kirchhoff modulus), for the analysed geometry does not have an impact on the change in the frequency or form of these natural vibrations (assuming the defined Young modulus). 

**Figure 5:**
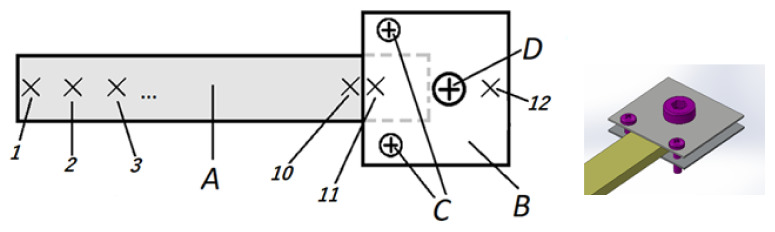
Diagram of the measurement: A—tested specimen; B—fixing metal element; C—fixing screws; D—exciter fixing screw; and 1–12—points of measurement.

In the next step, the specimen was attached to the vibration exciter and the above-described (Section 2) vibration scanning was carried out. The location of measurement points on the tested system’s geometry is presented in Figure 5. Measuring frequencies were in the range of 43–48 Hz with the scanning step of 0.1 Hz and 284–300 Hz with the scanning step of 0.2 Hz. An ambient temperature was maintained at the level of 23 ± 1 °C; air from the surroundings was gently blown onto the specimen by a fan. 

Measurements were performed at 10 points located 20 mm apart along the specimen’s center line (points 1–10) and at two points on the element holding the specimen (Point 11 and 12). Point 10 on the specimen and Point 11 on the fixture were as close to each other as possible, and close to the fixing element edge (about 2 mm). The measurements performed in the fixing system enabled an indirect assessment of stiffness through observations of the vibration phase angle between Point 11 and 12. 

Table 3 presents the results of the displacement amplitude measurement for all 12 points under analysis. The coordinates of the points defined in relation to the fixing screw (D) are given in the second row of the table. It can be seen that the first resonance frequency is included in the investigated range. However, a deeper analysis of the results indicates that precise determination of the resonant frequency is debatable. For the investigated vibration form (mode 1), the highest vibration amplitude occurs at the specimen’s far-end (which is the most distant from the support (Point 1)). The simplest pattern of finding the resonance frequency value (by finding the maximum amplitude in this place) gives the result of 45.5 Hz.

**Figure 6:**
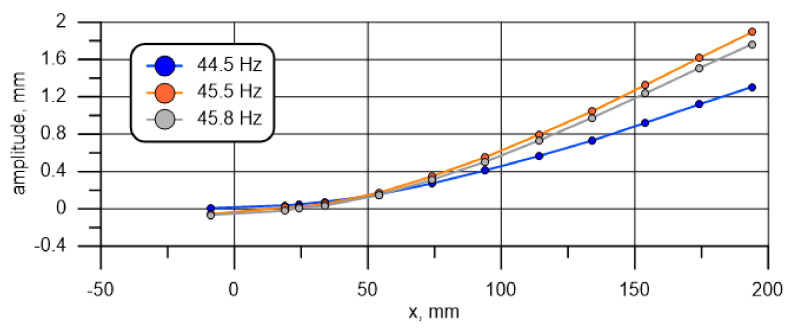
Amplitude at frequencies close to the first resonance frequency as a function of location.

Alternative approaches, i.e., finding the maximum vibration ratio between Point 1 and Point 12, 11, and 10, result in the respective values of 44.5, 45.5, and 45.8 Hz (marked). The curves illustrating the vibration forms for these three values are presented in Figure 6. 

It can be seen that the shape of the forms changes; the nodal point is shifted towards the specimen center with a rise in the base excitation frequency. Negative values represent a shift of 180° in relation to the other points (the value is rounded down or up to 0° (360°) or 180°). Because, under the adopted theoretical assumptions and using the constructed numerical model only the specimen’s protruding part is simulated, the most appropriate approach in this situation is to adopt a frequency for which the waveform node is located on the interface between the specimen’s tested part and the support as the natural frequency of mode 1. Considering the discrete character of the measurements, it is assumed that the point is located half way between measuring points 10 and 11. It follows from Table 3 that the lowest amplitudes for these two points, which at the same time result in the occurrence of the minimum of the approximating function between them (built on all 12 points of measurement), correspond to 45.6 Hz. This value is adopted as the first resonance frequency adequate for further consideration.

A similar procedure can be applied for the next resonance amplitude. The value determined in this way is 290.6 Hz if the criterion is the maximum amplitude of the end, which is the most distant point from the fixing screw, and 296.2 Hz if the waveform node is assumed on the specimen and fixture interface.

**Figure 7:**
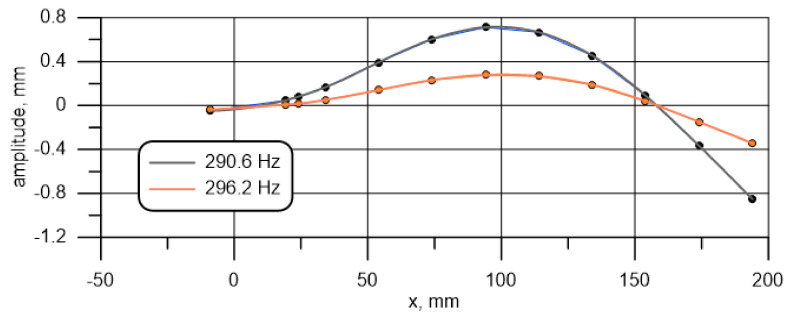
Amplitude at frequencies close to the second resonance frequency as a function of location.

Figure 7 illustrates the natural vibration forms for these frequencies. It should be noted that for the two frequencies, the point for which the approximated amplitude is zero, i.e., the point corresponding to the specimen cross-section that does not move vertically, but only rotates, is located at a position close to x=158 mm.

The difference between the node location for the two frequencies (Figure 7) is less than 0.5 mm and, considering the measurement accuracy, is of no practical significance. A higher vibration amplitude for the lower frequency does not necessarily mean a different location of the excited wave nodal point. It may just be due to the way in which energy is supplied to the oscillating system or characteristics of the exciter and the amplifier system. The value of 290.6 Hz was adopted for further consideration.

**Figure 8:**
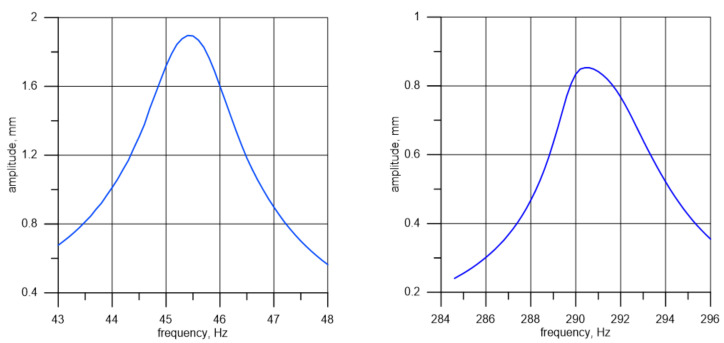
Amplitude depending on the vibration frequency for the first (**left**) and second (**right**) vibration form of specimen I.

Figure 8 presents the curves illustrating changes in the beam end amplitude, depending on the frequency in the resonance area of the first two bending modes. It can be observed that the curve representing the second mode is more asymmetric in relation to the extremum. This problem may be due to an unknown relation between the exciting force and frequency. It is unknown because, firstly, the characteristic of the sound card amplification, the amplifier, and the exciter is not perfectly linear, and secondly, because the specimen mass and motion affect the motion of the exciter. This characteristic has been corrected to a certain extent, but only for an exciter without a specimen attached to it. If the specimen is attached, especially if there is motion near resonance, additional inertia forces are introduced into the system. In turn, this has a significant impact on the motion of the exciter core and the forces generated within it. Modeling this phenomenon would require knowledge on the exciter’s internal structure (core geometry, number of cores, magnetic and electromagnetic field distribution, etc.) and the amplifier’s response to induced currents. The same phenomenon has, among others, an impact on the shape of the curves in Figure 8 and the amplitudes in Figure 6 and Figure 7.

**Figure 9:**
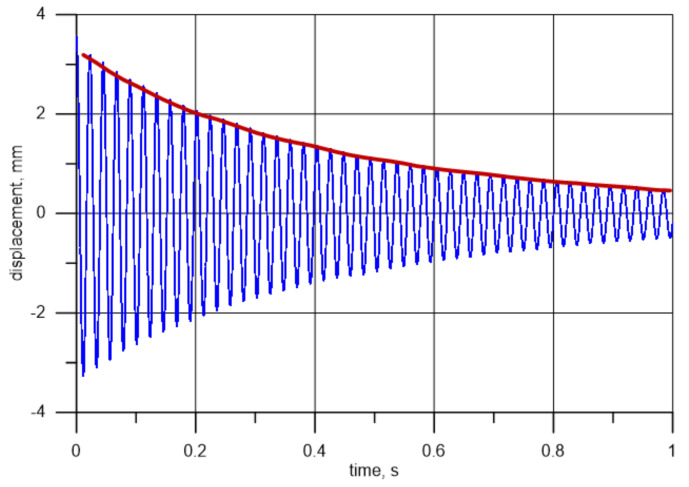
Displacement (distance) depending on selected time points for free vibrations with a decreasing amplitude for specimen I.

Apart from the measurements mentioned above, tests on the analysed element free vibration were also performed. For this purpose, the beam was statically bent by about 3.5 mm at its free end. The end was then released and the specimen motion was recorded using the optical displacement sensor employed previously. The sensor was placed at Point 1 (cf. Figure 5). The recorded changes in the vibration signal for the 197 × 20 × 6 mm beam are illustrated in Figure 9. The Fourier analysis performed for this data set indicates the frequency value of 44.77 Hz.

### 4.2. Evaluation of Vibration Damping

The measurement results presented in Figure 9 were used to evaluate vibration damping of the tested specimens. To this end, an algorithm that detects subsequent maxima of the history of changes in values of displacement in subsequent periods and then calculates the ratio between energy (V) in the oscillating element in a given cycle and in the cycle preceding it was created. Based on that and using slight transformations, it was possible to determine the loss factor in relation to a single cycle.

(7) $$\eta =\frac{\Delta V}{V}=\frac{V_{i-1}-V_{i}}{V_{i-1}\;}\;$$

**Figure 10:**
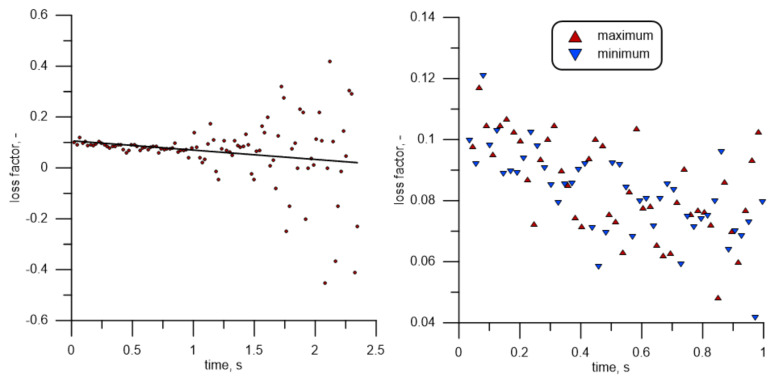
Loss factor depending on time (**left**) and a detailed view of the loss factor, depending on the time (**right**).

It was assumed that stored energy is proportional to the square of the amplitude, as in the case of a harmonic oscillator. The obtained results are shown in Figure 10 (left). The same results, but calculated on a different time scale and taking account of the division into values established using minima and maxima (for subsequent periods), are presented in Figure 10 (right). The average value of η for the first second of the measurements totals 8.4% for the function maxima shown in Figure 9, and 8.2% for the minima. With time, and as the amplitude decreases, the relative error, and consequently the spread of points around the average value, increases. For this reason, measurements should be limited to the initial phase of oscillations right after they are induced. Figure 10 illustrates changes in the relative energy drop in subsequent cycles.

The presented procedure is equivalent to matching a curve described by equation $Ae^{-\zeta \omega_{n}t}$ to measurement data [50] and establishing a specific damping ratio ζ as a loss factor per radian:

(8) $$\zeta \;=\;\frac{\eta \;}{4\;\pi \;}.$$

Factor 4 results from the fact that, at resonance, the loss factor is twice the loss damping ratio. Loss factor values lower than zero contradict the laws of physics. If such a value is found, it is an effect of measuring errors and indicates that the apparatus has an inadequate accuracy. This is particularly visible and significant at small amplitudes, where the relative error becomes unacceptably large. The red line in Figure 10 represents a straight line matched to measuring data. It can be seen that as the displacement amplitude decreases, so does the loss factor, which is due to the material’s smaller strains. Additionally, the aerodynamic interaction between the oscillating element and the environment decreases substantially. 

Figure 9 also shows the displacement amplitude history of Point 1 (red curve). It can be seen that at a free-end displacement of the order of 3.5 mm, after about 1 s, the amplitude falls below 1 mm. This explains the rising relative error mentioned above. The presented methods deliver loss at every one period, contrary to matching the $Ae^{-\zeta \omega_{n}t}$ curve. This also enables an analysis of the change in the loss factor during each period, together with the change in the amplitude (and time). It has to be mentioned that the samples are collected at a much higher frequency compared to the vibrations. Even in an extreme situation, where the real extremum is exactly in the middle of the collected samples, the deviation of the determined maximum is not bigger than the sensor resolution.

### 4.3. Phase Angle Analysis

The developed test stand enables phase angle measurements using a single displacement sensor, as described above in Section 2.2. The phase shift is herein understood as relative displacement phase between points of measurement close to the specimen support (10, 11, or 12) and Point 1 (beam free end). The phase shift is determined using the same measurement results described above (cf. Table 3). The data are additionally normalized to values of 0–360, where 0° denotes synchronized motion, and positive values correspond to the beam end “delay”. The curve illustrating the phase shift between points 1 and 10 is adopted for further consideration, as this curve best simulates the above-assumed beam eigenfrequency. 

The phase angle can be determined analytically using the following relation [50]:

(9) $$\theta_{\zeta }\left(\omega \right)=tan^{-1}\;\frac{2\;\zeta r}{1-\;{r^{2}}^{\prime }}\;$$

where $r=\omega /\omega_{n}$ is the ratio of forced $\left(\omega \right)$ and natural $\left(\omega_{n}\right)$ frequency.

**Figure 11:**
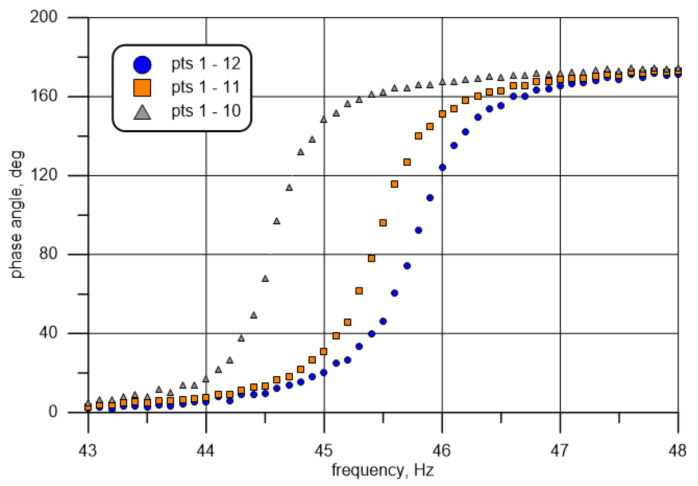
Frequency-dependent phase angle between measuring Point 1 and Point 10—gray, Point 11—orange, and Point 12—blue.

Based on Equation (9), it is possible to draw a theoretical curve and compare it to the measuring curve (frequency-dependent changes in the phase angle) for known values of the damping ratio and natural frequency. It follows from Equation (9) that a change in natural frequency involves a change in the location of the point where the curve runs through the value of 90 degrees, and a change in damping affects the curve slope or flattening. A rough analysis of the data marked in orange squares in Figure 11 (the phase shift between points 1 and 11) indicates that the natural frequency is close to 45.4 Hz, i.e., $\omega_{n}=285.3\;s^{-1}$ (phase angle of 90°). The values were established with a better accuracy using the Microsoft Excel Solver tool. For a frequency of about 45.4 Hz from the range of 44.7–46.1 Hz with the resolution of 0.1 Hz, the approximating function defining the history of frequency-dependent changes in the phase angle was determined. This was achieved by minimizing the functional $\delta_{\zeta }\left(\omega \right)$ of the sum of squares of differences in the measured ($\theta_{mi}$) and theoretically established ($\theta_{ti}$) phase angle values for each of the fifteen frequencies (points) under analysis (frequency closest to the 90° in the middle):

(10) $$\delta_{\zeta }\left(\omega \right)=\frac{1}{n}\;\overset{n}{\underset{i=1}{\sum }}{\left(\theta_{ti}-\theta_{mi}\right)}^{2}\to min.$$

The value obtained in this manner was the minimization criterion for the “solver” function. The initial values for the described procedure algorithm were assumed to be η=0.07 (value close to the one obtained from the testing of individually excited, free vibration) and $f_{n}=45.4\;\mathrm{Hz}$. This produced values of η=0.082 and $f_{n}=45.44\;\mathrm{Hz}$, which were then adopted as exact resultant values of the determined quantities. 

**Figure 12:**
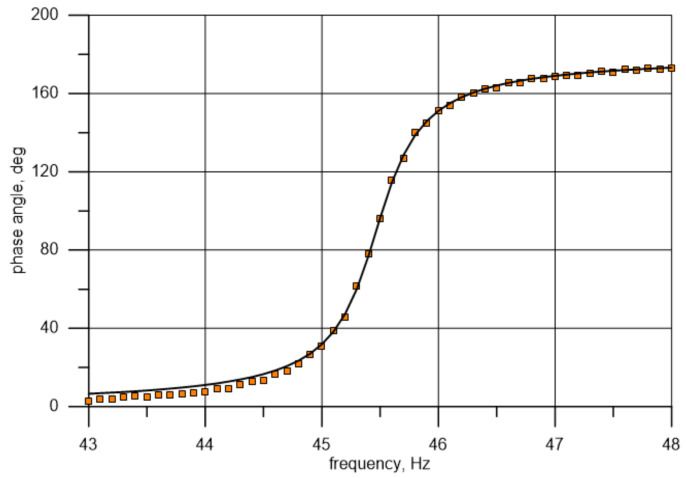
Comparison of the phase angle obtained from measurements and its theoretical distribution after eigenfrequency and damping were matched to natural frequency and damping values (1st bending mode).

**Figure 13:**
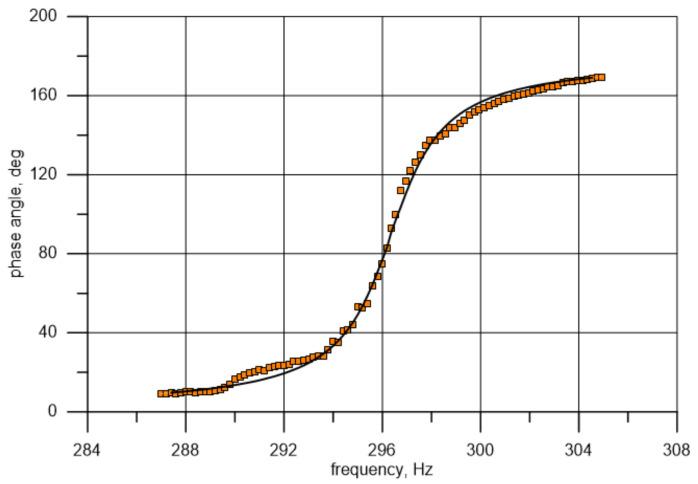
Comparison of the phase angle obtained from measurements and its theoretical distribution after eigenfrequency and damping were matched to natural frequency and damping values (2nd bending mode).

A similar procedure for the phase angle analysis was adopted for the next natural vibration form corresponding to the frequency of about 296 Hz. The theoretical curve was matched based on data from the range of 292–300 Hz. The obtained values of the loss factor and natural frequency were η=0.057 and $f_{n}=296.3\;\mathrm{Hz}$, respectively. The matching of the curves, together with the points representing the results of measurements, are presented graphically in Figure 12 and Figure 13. For the used test bench, the uncertainty of the measured phase angle values (for one point) is approximately 0.5° for about 45 Hz and changes proportionally with the frequency. The uncertainty of the measured amplitude (for one point) is approximately 0.001 mm and the uncertainty of the measuring frequency is insignificant. 

The above procedure was adopted for the other two specimens. Table 4 below presents the results obtained for all three beams (specimens I, II, and III). 

For the first two specimens, which were longer, the data were collected for three different excitement levels. This was achieved by changing the amplification of the channel controlling the vibration exciter, which is expressed quantitatively by the maximum achievable amplitude (the highest amplitude in the entire history at this test point) and described as the “maximum amplitude”. In the case of specimen I, the amplitudes were 0.95, 1.89, and 3.71 mm for the first bending mode. For the first vibration form, no matter which criterion was adopted (the maximum amplitude of Point 1 or the ratio between the amplitude at Point 1 and Point 11), the result was the same. The next field for a given data set marked as the “determined frequency (max amplitude)” defines the resonance frequency found by establishing the amplitude maxima (measuring point 1) depending on the frequency. The next two fields below show the results obtained from the phase angle shift between points 1 and 11. In the case of the next bending mode, for a frequency of about 290–300 Hz for specimen 1, the values are presented in the same way as for the first vibration form. Two frequencies are given in the table for each element. The first was established based on the amplitude maximum value at Point 1 and the second was determined from the maximum ratio between the amplitude at Point 1 and in Point 11. For specimen III, due to the beam’s higher eigenfrequencies and, consequently, smaller displacement amplitudes that could be achieved, a smaller amount of data was collected. The test stand structure, including the kind of input function and measured quantities (displacement), guarantees a higher resolution and measuring accuracy for low frequencies. In general, a rise in frequency involves a decrease in the accuracy of determination of individual indices in the presented configuration of the test bench.

The result marked with an asterisk (*) defines the specimen end amplitude estimated based on measurements performed at the element’s other points (2, 3, 4, …) knowing the beam deflection shape. The result itself is beyond the measuring sensor range (amplitude of 5 mm for a symmetrical configuration).

Based on the data listed in Table 4, it can be concluded that oscillations are damped more intensively for bigger amplitudes, which can be seen in Figure 10. This may be related to another behavioral pattern of the material: Its nonlinearity for high stress and strain values, or to resistance of the air surrounding the specimen as air is pumped along the longer side due to the specimen’s motion. At this stage, it is impossible to carry out a quantitative analysis of the impact of individual phenomena. Further numerical, analytical, and experimental investigations are required.

However, using the presented measuring data, it is possible to determine the material’s elastic modulus E. Using relation (6) defining the prismatic cantilever beam’s natural vibration, it can be stated that the eigenfrequency is proportional to the square root of E.

(11) $$f\;\sim \;\sqrt{E}$$

Based on this relation and using the data obtained from numerical calculations, the material stiffness value in the model taken from the material technical data sheet (2852 MPa) can be corrected. The elastic modulus values determined from the measurements are listed in Table 5.

Differences of about ± 10% between individual specimens can be noticed for the first mode. The situation is similar for the second mode, but not for specimen III, which is characterized by the highest stiffness of all the tested elements. The elastic modulus value is very different in that case. This may be due to frequency-dependent changes in the material elastic properties. It may also be the effect of the significant relative error in amplitude determination (very small amplitude of the input function at the sensor constant error) and higher natural frequency (fewer points in time domain mapping the oscillating motion).

## 5. Discussion

Based on numerical calculations, it was found that the analysed damping values (determined from the history of changes in the phase angle) have little significant impact on natural vibration values, which is in agreement with theory ($\omega_{d}=\omega_{n}\sqrt{1-\zeta^{2}}$). The square root value for the observed instances of damping exceeds 0.99.

The values obtained from tests where free vibrations were excited for the other elements are listed in Table 6. The presented data illustrate the loss factor for amplitude values higher than 0.5 mm. Several tests were performed for each element. Their number is given in the table and the loss factor determined in each test never differed by more than 0.01 from the element average. 

The results presented in Table 6 suggest the diversity of intense values of the material stiffness with the specimen thickness. This may be due to stresses and material defects arising during the printing process and related to the manufacturing technology and cooling process. The phenomenon requires further analysis. It may also be an effect of the interaction and relative motion of the material layers. 

Certain phenomena characterizing the developed and applied methodology, particularly the measuring and research methodology, were observed during the testing. Firstly, temperature has a substantial impact on the observed indications, and the resonance frequency values are especially affected. The temperature during the testing was maintained at the level of 23 ± 1 °C, but additional tests showed considerable variability of the resonance frequency (of the order of a few/several percent) in the temperature range of 18–32 °C. 

The presented vibration testing can be used to determine the elastic modulus of an element made with AM technology. Apart from the base material, the elastic modulus value depends on the infill structure, the orientation of added layers, the infill percentage, and the infill quality. The most important factors are the quality of the layer-to-layer adherence/joint cohesion and possible geometrical discontinuities arising during the printing process. The nozzle temperature is probably also essential, but this requires further analyses. When determining the elastic modulus by means of the method presented herein, care should be taken to appropriately select the tested element’s geometrical dimensions to meet the characteristics of the applied measuring apparatus. First and foremost, an opportunity should be created to ensure an adequately high amplitude to minimize the relative measuring error. Another important factor is the ratio between the specimen resonance frequency and the sampling frequency. The smaller the ratio, the more precise the vibration simulation. As a result, the stiffness will be established with a much better accuracy. 

The tested element heating due to the work of friction forces inside the material may also have been significant in the testing described herein. To minimize the problem, it was made sure that, during each measurement (especially in the range of resonance frequencies), no temperature gradient higher than 1 °C was created. It was done by thermovision infrared camera. The susceptibility to this effect is strongly dependent on the tested element’s geometry, amplitudes, input function forces, and specimen cooling. 

**Figure 14:**
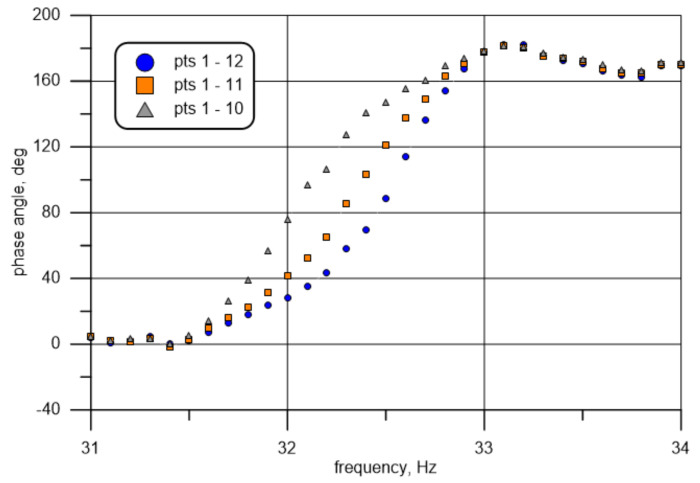
Frequency-dependent phase angle between measuring Point 1 and Point 10 for too short a run-up time: Point 10—gray; Point 11—orange; and Point 12—blue.

Another phenomenon observed during the measurements worth noting is the impact of the test run-up time, i.e., the period from the moment the exciter is activated to the start of data acquisition. For the time of half a second, the values gathered and presented in the manner described above (cf. Figure 11) are shown in Figure 14 (specimen II). A local maximum and minimum can clearly be seen close to 33.2 and 33.8 Hz, respectively. Other characteristic deformations of the curves can also be noticed in the chart. They re-appeared when the tests were repeated and they are visible for all three basic points under analysis (10, 11, and 12). The phenomenon became weaker with an increase in the run-up time. This leads to the probable cause of the curve deformation mentioned above. It may be due to acoustic beats caused by the wave excited by the exciter’s continuous motion (quasi-steady state) and the excited wave frequency when motion starts (transient-state condition). All the results presented above were obtained for a sufficiently long run-up time. According to the damping characteristic described above (roughly, a constant part of energy is lost in every period of motion), the time depends on the analysed frequency. 

When performing numerical calculations and comparing their results to experiments, attention should be focused on the adopted model assumptions, which are usually a simplification. This particularly concerns the support conditions, which may differ from real conditions in many ways. One example is a support which is not stiff enough. Apart from its translational motion, slight rotation also occurs.

## 6. Summary and Conclusions

This paper presents a novel procedure for vibration measurements using a single measuring sensor. An optical displacement sensor with the measuring range of 10 mm is introduced. Such an approach limits the measuring apparatus costs and enables phase angle measurements for very small elements, where, for reasons related to the element dimensions, the standard use of two sensors is impossible. Also shown are methods of interpretation and usefulness of the data collected in this way. The data make it possible to determine selected mechanical parameters, such as the density, dynamic modulus of linear stiffness, shear modulus, material internal damping, etc. The proposed method enables measurements of damping and the resonance frequency in cases where the history of the frequency-dependent input function force is unknown. 

An analysis of selected dynamic properties of elements made with 3D printing technology was conducted. To achieve a comprehensive assessment of the obtained values and to estimate measuring errors, it is necessary to repeat tests for a bigger number of specimens and perform a statistical analysis. It should be emphasized that the presented results depend on a large number of variables, e.g., the printing temperature, measurement ambient temperature, course of the printing process, etc. It seems that, if resonance frequencies are selected appropriately, the proposed method makes it possible to carry out detailed and reliable tests. The key issue is recording the displacement amplitude during the testing as accurately as possible, which depends on the measuring sensor’s quality and the sampling frequency. The presented method can also be used with velocity and acceleration sensors. With appropriately selected vibration frequencies, this should improve the testing accuracy. The testing presented in the paper made it possible to record displacement in the range of 100–1600 measurements per period, i.e., with a phase accuracy of about 3.5° to about 0.2°. Lower sampling frequencies will worsen the accuracy, e.g., due to “missing out” the displacement extremum, to the poorer matching of selected approximating functions or to the aliasing. A reduction in the sampling frequency decreases the resolution and phase angle measurement accuracy for the determined waveform. 

This paper presents a method of phase angle measurement using a single sensor enabling the determination of selected mechanical properties, in particular, the resonance frequency and damping in cases where functions of the quantitative description of the vibration exciter (force and base excitation) are not constant and their history is unknown. This makes it possible, for example, to use the system of the sound card, amplifier, and oscillator with different characteristics, which are also modified due to the dynamic impact of the fixed specimen. 

It was found from the testing results that, in the analysed cases, the model of the loss of a constant part of the oscillating system energy described damping fairly well. No dramatic variations were observed with a change in the vibration frequency in the range of 30–300 Hz. Other aspects worth noting are the support stiffness and the stiffness of the entire exciter system. The support impact was substantially eliminated by an adequate selection of the phase measurement points. Nevertheless, the support stiffness may have affected the results to a certain extent. The results indicate that the metal fixture (the support) displacement was slight, but even small up-and-down motion non-synchronized in phase may, in the case of the described geometry, have a significant effect on the specimen behavior. This problem needs to be analysed further. The selected resonance occurrence criterion (minimization of the amplitude on the specimen-support interface) minimizes the error resulting therefrom.

## Figures and Tables

**Table 1:** Specimen data.

Element	Length mm	Width mm	Thickness mm	Mass g	Density g/cm^3^
I	197.0	20.0	6.0	29.16	1.24
II	197.0	20.0	4.0	19.55	1.24
III	117.0	20.0	4.0	11.55	1.24
				avg. ->	1.24

**Table 2:** Vibration modes of specimen I.

Mode	1	2	3	4	5	6	7	8	9
Freq., Hz	46.6 *	153.5	290.6 *	698.2	807.4 *	911.3	1565.0 *	2102.9	2134.4

**Table 3:** Measurement results of specimen I.

pt. Number →	1	2	3	4	5	6	7	8	9	10	11	12
position x mm →	194	174	154	134	114	94	74	54	34	24	19	−9
**Freq., Hz ↓**	Amplitude, mm ↓											
**44.2**	1.116	0.954	0.787	0.628	0.489	0.354	0.238	0.139	0.073	0.050	0.040	0.012
**44.3**	1.170	1.007	0.826	0.657	0.511	0.372	0.249	0.143	0.073	0.050	0.039	0.010
**44.4**	1.240	1.056	0.873	0.694	0.543	0.391	0.260	0.147	0.074	0.049	0.038	0.008
**44.5**	1.307	1.119	0.921	0.732	0.565	0.411	0.272	0.153	0.075	0.048	0.037	0.007
**44.6**	1.380	1.182	0.968	0.769	0.596	0.432	0.285	0.158	0.075	0.048	0.036	0.009
**44.7**	1.475	1.252	1.026	0.817	0.629	0.453	0.298	0.164	0.075	0.046	0.033	0.012
**44.8**	1.555	1.329	1.091	0.858	0.665	0.475	0.312	0.168	0.075	0.045	0.031	0.016
**44.9**	1.638	1.399	1.148	0.908	0.699	0.497	0.324	0.173	0.073	0.043	0.028	0.022
**45.0**	1.720	1.461	1.196	0.949	0.729	0.518	0.336	0.177	0.074	0.040	0.025	0.027
**45.1**	1.789	1.527	1.248	0.987	0.753	0.535	0.344	0.180	0.068	0.036	0.021	0.034
**45.2**	1.844	1.571	1.289	1.019	0.775	0.548	0.350	0.181	0.064	0.033	0.018	0.040
**45.3**	1.878	1.603	1.311	1.040	0.786	0.555	0.353	0.179	0.061	0.028	0.015	0.046
**45.4**	1.896	1.615	1.326	1.051	0.792	0.556	0.352	0.176	0.057	0.023	0.013	0.051
**45.5**	1.893	1.613	1.326	1.045	0.793	0.552	0.347	0.171	0.052	0.019	0.013	0.056
**45.6**	1.869	1.595	1.304	1.036	0.777	0.542	0.338	0.164	0.046	0.015	0.014	0.060
**45.7**	1.828	1.561	1.283	1.008	0.757	0.524	0.325	0.156	0.041	0.013	0.017	0.064
**45.8**	1.764	1.506	1.237	0.977	0.731	0.504	0.310	0.146	0.035	0.011	0.020	0.066
**45.9**	1.689	1.442	1.187	0.936	0.697	0.481	0.293	0.134	0.030	0.011	0.024	0.068
**46.0**	1.601	1.372	1.130	0.891	0.660	0.452	0.274	0.124	0.023	0.013	0.027	0.069
**46.1**	1.515	1.295	1.065	0.840	0.623	0.425	0.255	0.113	0.018	0.015	0.029	0.069
**46.2**	1.427	1.219	1.004	0.790	0.586	0.399	0.236	0.102	0.014	0.017	0.031	0.069

**Table 4:** Vibration data for investigated specimens.

		Element	I 178 × 20 × 6 mm	II 178 × 20 × 4 mm	III 98 × 20 × 4 mm
		calculated frequency I, Hz	46.616	31.115	100.96
		calculated frequency II, Hz	290.59	194.51	627.61
**First bending mode**	Amplification 1	maximum amplitude, mm	0.95	1.56	0.36
		determined frequency (max amplitude), Hz	45.6	32.3	97.1
		determined frequency (phase shift), Hz	45.52	32.31	97.06
		loss coefficient, -	0.065	0.052	0.06818
	Amplification 2	maximum amplitude, mm	1.89	3	0.87
		determined frequency (max amplitude), Hz	45.5	32.3	97.5
		determined frequency (phase shift), Hz	45.44	32.3	97.52
		loss coefficient, -	0.082	0.06	
	Amplification 3	maximum amplitude, mm	3.71	5.9 *	
		determined frequency (max amplitude), Hz	45.4	32.2	
		determined frequency (phase shift), Hz	45.41	32.29	
		loss coefficient, -	0.083	0.074	
**Second bending mode**	Amplification 1	maximum amplitude, mm	0.18	0.7	0.36
		determined frequency (max amplitude), Hz	291.2/296.0	187/193.2	501.8/511.8
		determined frequency (phase shift), Hz	297.8	193.04	
		loss coefficient, -	0.053	0.066	
	Amplification 2	maximum amplitude, mm	0.85	1.21	0.6
		determined frequency (max amplitude), Hz	290.6/296.2	186.4/193.2	499.4/512
		determined frequency (phase shift), Hz	296.3	193.12	
		loss coefficient, -	0.07	0.068	
	Amplification 3	maximum amplitude, mm	1.32	1.76	
		determined frequency (max amplitude), Hz	290.2/295.4	185.6/192.6	
		determined frequency (phase shift), Hz	295.2	192.67	
		loss coefficient, -	0.07	0.075	

**Table 5:** Elastic modulus results.

Element	Mode	Frequency, Hz		E, MPa
		Calculated	Measured	
I	1	46.6	45.4	2705
II		31.1	32.3	3073
III		101.0	97.3	2649
I	2	290.6	296.4	2968
II		194.5	192.9	2806
III		627.6	512.0	1898

**Table 6:** Loss factor determined experimentally.

Specimen	I	II	III
Loss factor	0.085	0.067	0.087
Number of tests	2	3	8

## Data Availability

The data presented in this study are available on request from the corresponding authors.
